# Survey of the Diagnostic Retooling Process in National TB Reference Laboratories, with Special Focus on Rapid Speciation Tests Endorsed by WHO in 2007

**DOI:** 10.1371/journal.pone.0043439

**Published:** 2012-08-24

**Authors:** Sanne C. van Kampen, Linda Oskam, Coosje J. Tuijn, Paul R. Klatser

**Affiliations:** Royal Tropical Institute, Royal Tropical Institute Biomedical Research, Amsterdam, The Netherlands; The Australian National University, Australia

## Abstract

**Background:**

Successful integration of new diagnostics in national tuberculosis (TB) control programs, also called ‘retooling’, is highly dependent on operational aspects related to test availability, accessibility and affordability. This survey aimed to find out whether recommendations to use new diagnostics lead to successful retooling in high TB endemic countries, using immunochromatographic tests (ICTs) for TB culture speciation as a case study. ICTs are recommended to accurately confirm the presence of bacteria of the *Mycobacterium tuberculosis* complex in liquid culture isolates.

**Methods and Findings:**

Questionnaires were sent to national TB reference laboratories (NRLs) in 42 high TB endemic countries to address their access to information on ICT implementation, logistics related to availability, accessibility and affordability of ICTs, and testing algorithms. Results from 16 responding countries indicated that half of the NRLs were aware of the contents of WHO guidance documents on liquid culture and ICT implementation, as well as their eligibility for a negotiated pricing agreement for ICT procurement. No major issues with availability and accessibility of ICTs were raised. When asked about testing algorithms, ICTs were not used as stand-alone or first test for TB culture identification as recommended by WHO.

**Conclusions:**

The low response rate was a limitation of this survey and together with NRLs managers' unawareness of global guidance, suggests a lack of effective communication between partners of the global laboratory network and NRLs. TB tests could become more affordable to high TB endemic countries, if the possibility to negotiate lower prices for commercial products is communicated to them more successfully. NRLs need additional guidance to identify where available technologies can be most usefully implemented and in what order, taking into account long-term laboratory strategies.

## Introduction

Tuberculosis (TB) is still a leading cause of death in low- and middle-income countries. One major challenge is to rapidly and effectively diagnose TB in an early stage of disease [1]. In 2007, WHO recommended the use of liquid culture for accelerated growth of bacilli from sputum specimens to improve timely TB diagnosis and drug-susceptibility testing (DST) [2]. In comparison to using solid media for TB culture, liquid broth is less selective for growth of nontuberculous mycobacteria (NTM) and it is therefore essential to differentiate between species of the *Mycobacterium tuberculosis* (MTB) complex and NTM. While various speciation methods can be used, WHO recommends the use of lateral flow immunochromatographic tests (ICTs). These so called rapid speciation assays are able to accurately confirm the presence of a member of the MTB complex within fifteen minutes, are easy to perform and affordable to most TB control programmes [3]. In short, a liquefied culture specimen is placed onto a nitrocellulose strip in a cassette containing labeled antibodies specific for TB antigen. If TB antigens are present, they bind to the antibodies which are then immobilized on the strip and this positive binding is visualized by a coloured band. ICTs for speciation are preferred to standard biochemical tests, such as nitrate reduction and *p*-nitrobenzoic acid assays, because they can ensure rapid turn-around time of confirmed liquid culture results for both the technician and the patient and are less expensive in routine settings [4]. ICTs are preferred to line probe assays – where DNA isolation and amplification steps are followed by detection of MTB complex DNA by hybridization to specific oligonucleotide probes bound on a cellulose strip - because they are less labour intensive, less time-consuming and less costly [5].

**Figure 1:**
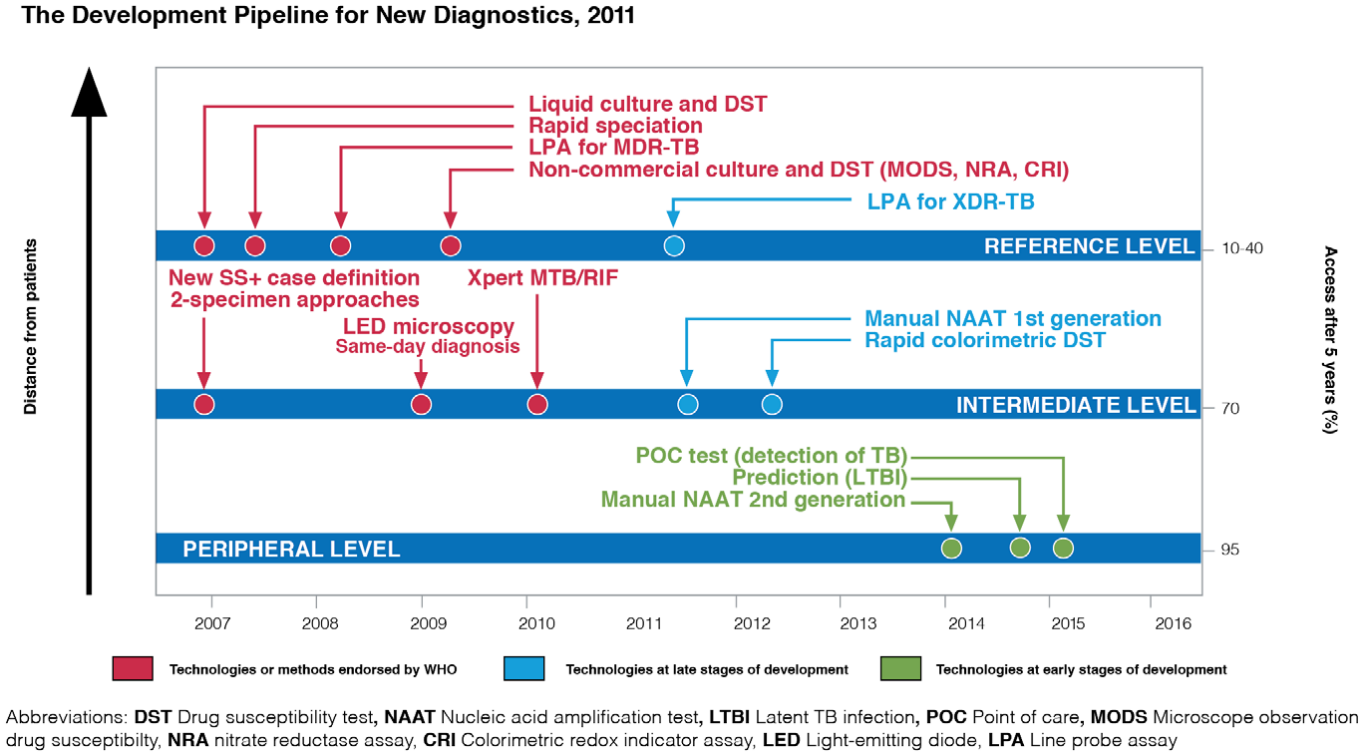
Diagnostic development pipeline. The status of the pipeline for new diagnostics for tuberculosis in July 2011 and the placement of liquid culture and rapid speciation tests within it. Reproduced from ‘Global tuberculosis control: WHO report 2011’ with permission.

Figure 1 illustrates WHO endorsement and placement of both liquid culture and rapid speciation assays within the diagnostic development pipeline [6]. Since WHO recommended to use liquid culture systems and ICTs for speciation in national TB reference laboratories (NRLs) in low- and middle-income countries, ever more countries have introduced these techniques into their national TB laboratory network. The process of introducing and integrating new diagnostic tools into national TB control programmes (NTPs) is referred to as diagnostic retooling. In recent years there has been a global realization that diagnostic retooling can only be successful if the gap between global policy and local adoption of new tools is addressed. In particular, the Stop TB Partnership subgroup for ‘Introducing New Approaches and Tools’ (INAT) has worked to obtain information from NTPs and other implementing partners on the challenges in evaluating, adopting, introducing and implementing new tools or approaches, and formulated international guidance accordingly [7]. Also, the ‘Technology, Research, Education and Technical Assistance for Tuberculosis’ (TREAT TB) initiative of the Union is promoting research on comprehensive TB diagnostic approaches followed by translation of evidence into country-relevant policy and practice, especially through their Diagnostic Tools Initiative [8]. A recent paper by Van Kampen *et al.* highlighted the status of first-phase diagnostic retooling in high burden TB countries in 2009 and some of the problems faced by NTP managers during adoption of diagnostic methods newly recommended by WHO [9]. It is important to further explore the subsequent stages of retooling, namely integration of new diagnostics into routine laboratory practices, and evaluation of their use within national TB laboratory networks. Successful integration of new tools is highly dependent on operational aspects related to test availability, accessibility and affordability.

The aim of this survey was to find out whether recommendations for the use of new diagnostics result in successful retooling in high TB endemic countries, using ICTs for liquid culture speciation as a case study. Retooling national TB laboratory networks with ICTs is an attractive case study because of various factors: the stand-alone nature of the test, relative lack of accessories required, simple training, simple storage requirements, readily identified end-users (limited to culture and reference laboratories), limited number of known suppliers, no known counterfeits, and no commonly-used in-house equivalents. Currently there are three known suppliers of ICTs for MTB complex identification - all using TB specific anti-MPT64 monoclonal antibodies attached to a strip-, namely Tauns in Japan producing the Capilia TB-Neo assay, SD Bio Standard Diagnostic in India and South Korea producing the TB Ag MPT64 Rapid assay, and BD Diagnostics in the USA producing the BD MGITTM TBc Identification Test [10], [11], [12]. WHO recommends countries implementing liquid culture and ICTs to set up commercial sales contracts with manufacturers to ensure continuous availability and accessibility of test equipment and consumables at reasonable costs. At this time, it is not well known what type of commercial sales contracts NTPs have with ICT producers. Further, favorable pricing schemes for the procurement of BD products are facilitated by the Foundation for Innovative New Diagnostics (FIND) in Geneva for the public and private not-for-profit sector in high endemic countries as listed on their website, through which the BD MGITTM TBc Identification Test is available for $2.90 per piece ex works [13]. It is not well documented whether these countries make use of the favorable pricing agreements and whether there are additional negotiated pricing systems in place for other countries or for other speciation tests.

## Methods

A detailed survey of retooling activities was conducted in high TB endemic countries. Fifty countries were selected comprising nine high-burden TB countries, 14 high-burden multidrug-resistant (MDR-)TB countries, 13 high-burden TB and MDR-TB countries, nine countries that participated in the EXPAND-TB project [14], and an additional five countries with a high TB prevalence, and geographically covering all WHO regions (16 from AFRO, 15 from EURO, seven from SEARO, four from AMRO, four from EMRO, and four from WPRO). Contact details of NRL managers of 42 countries were obtained from the Laboratory Directory on the website of the Global Laboratory Initiative (GLI) TB Lab Network [15], as well as communication with colleagues of the global laboratory network and relevant NTP managers. Questionnaires were sent in February 2011 by email with a request to complete and return the document within five weeks. The questionnaire contained 31 closed and five open questions aimed at addressing the following:

1. Do NRL managers in high endemic countries have access to information related to liquid culture and ICTs for speciation (WHO Expert Group Meeting Report, Retooling Task Force Checklist, and Global Laboratory Initiative Roadmap)? (five closed questions)
2. How do logistics influence NRL's access to ICTs, including procurement, distribution, and remaining shelf-life of tests? (six closed questions)
3. How adequate is internal forecasting, stock management and storage procedures to avoid stock-outs and wastage of ICTs in NRLs? (seven closed questions)
4. Is the model of negotiated pricing resulting in access to affordable diagnostics for the end-user of ICTs? (five closed questions, two open)
5. What are the diagnostic algorithms in which ICTs for culture speciation are routinely used in high endemic countries? (nine closed questions, two open)

To address question 1, electronic files on implementing new TB diagnostics and retooling processes were sent to NRL managers. Their ability to receive and open three electronic documents as well as their previous awareness of the contents was assessed. To address questions 2, 3 and 4, NRL managers were asked about their experiences with introducing ICTs for culture speciation into their TB laboratories and especially with test procurement and delivery, test storage management, and the availability of pricing schemes for procurement. Storage management includes keeping stock cards to record the number of tests in storage and anticipate timely order placement for new tests (‘internal forecasting’) to avoid running out of tests/supplies (‘stock-out’); recording temperature in storage rooms to avoid spoiling of tests; and recording test expiry dates in order to use tests within the time they can meet specified requirements (‘shelf-life’). To address question 5, NRL managers were asked about diagnostic algorithms in which ICTs were used in routine laboratory practice. Data entry and analysis was performed using Epi Info (CDC, 2008).

## Results

### Access to information on retooling with liquid culture and ICTs for speciation

Contact details of 28 NRL managers were derived from the GLI website, 14 through communication with colleagues and relevant NTP managers, and eight of whom contact details could not be found. Of the 42 NRL managers that were contacted (Table 1), 16 (38%) returned a filled out questionnaire. From the WPRO region 50% (2/4) responded, from AFRO 38% (6/16), from EURO 33% (5/15), from EMRO and AMRO 25% (1/4), and from SEARO 14% (1/7).

**Table 1:** Forty-two countries that were sent a questionnaire separated by high-burden of TB, high-burden of multidrug-resistant (MDR-)TB, high TB prevalence, and participation in the EXPAND-TB project.

High-burden TB	High-burden MDR-TB	EXPAND-TB	High TB prevalence
Mozambique	Belarus	Senegal	Malawi
Zimbabwe	Estonia	Djibouti	Dominican Republic
Afghanistan	Latvia	Cameroon	Egypt
Cambodia	Lithuania	Cote d'Ivoire	Nepal
Thailand		Lesotho	Lao People's DR
Brazil		Swaziland	
Kenya *		Zambia	
Uganda *		Haiti	
UR of Tanzania *			
South Africa			
Nigeria			
Pakistan			
China			
Philippines			
	Kazakhstan		
	Kyrgyz Republic		
	Republic of Moldova		
	Tajikistan		
	Ukraine		
	Uzbekistan		
Democratic Republic of Congo			
Viet Nam			
Ethiopia			
Bangladesh			
India			

* These countries also participated in the EXPAND-TB project.

In order to assess their access to and awareness of guidelines on implementing liquid culture systems and ICTs for speciation, the participants were sent the following key electronic documents:

1. *Use of liquid TB culture and drug susceptibility testing (DST) in low and middle income settings. Summary report of the Expert Group Meeting on the use of liquid culture media*, WHO, 26 March 2007 [2],
2. *Checklist of key actions for the use of liquid media for culture and drug susceptibility testing (DST)*, Retooling Task Force of the Stop TB Partnership (presently referred to as ‘Innovative New Approaches and Technologies Work Group’), February 2008 [16], and
3. *A roadmap for ensuring quality tuberculosis diagnostics services within National Laboratory Strategic Plans*, Global Laboratory Initiative, 20 January 2010 [17].

Twelve of 16 respondents (75%) were able to open and view all three electronic documents, while four indicated not to be able to open any. One NRL manager that was not able to open the guiding documents provided no more answers after these first questions, thus further analysis was based on answers from 15 respondents. Eleven of 15 (73%) countries were using liquid culture systems for TB diagnosis – mostly the Mycobacteria Growth Indicator Tube (MGIT) assay – in their laboratory networks: two of them had used the Expert Group Meeting report to help in guiding implementation and three had used GLI's roadmap. Eight of 15 respondents (53%) were aware of the most important laboratory and policy requirements for liquid culture system introduction as described in the Expert Group Meeting report, while seven said to be unaware of some specific issues, notably the need for laboratories to establish a commercial sales contract and customer support plan with manufacturers. Seven (47%) NRL managers were unaware that WHO had officially recommended the use of ICTs to identify MTB complex in culture isolates.

### Logistics of access to ICTs for culture speciation

Eleven of 15 NRL managers performed liquid culture, of which eight (73%) had introduced ICTs for speciation in their laboratory. Two countries without liquid culture also used ICTs, probably to identity bacterial growth on solid media cultures (Table 2). Five were using TB Ag MPT64 Rapid Assay, four used Capilia TB-Neo, and two used BD MGITTM TBc Identification Test. The three laboratories that had not introduced ICTs while performing liquid culture provided as reasons: 1) waiting for official test evaluation and 2) lack of national decision. Liquid culture systems were introduced into NRLs as early as 1990, while rapid speciation assays were implemented from 2008 onwards. Out of ten NRLs using rapid speciation tests, five (50%) received ICT kits directly from non-governmental donors or other funding agencies and two (20%) received direct funding from such organizations to procure ICTs. When new ICTs were ordered from the local distributor, NRLs typically had to wait for five weeks before tests arrived at the laboratory. The remaining shelf-life of the assays was on average eight months (35 weeks). Four NRL managers indicated that temperature was not recorded during transportation of test kits to their laboratory; three said that this was done; and three others did not know whether this was done.

**Table 2:** Procurement and logistics of immunochromatographic tests for identification of bacteria of the *Mycobacterium tuberculosis* complex in positive culture isolates in national TB reference laboratories of 15 high endemic countries.

NRLs	Liquid culture used	ICT used	Type of ICT	Stock-outs/year	Test expiry	Delivery time (weeks)	Shelf-life (weeks)	Orders placed/year	Records of test usage	Direct from donor	Funds from donor	Sales contract	Eligible for negotiated pricing	On FIND list^1^
A	Y	Y	Tauns	2	Y	16	26	2	Y	Y	n.a.	N	N	Y
B	Y	Y	Tauns & SD	DNK	DNK	-	-	-	Y	Y	n.a.	Y	DNK	Y
C	Y	Y	SD	0	Y	4	52	2	Y	Y	n.a.	N	DNK	Y
D	Y	Y	Tauns	0	DNK	4	12	4	Y	Y	n.a.	N	Y	Y
E	Y	Y	BD	0	DNK	4	6	2	Y	N	N	DNK	Y	N
F	Y	Y	BD^2^	0	N	2	43	2	Y	N	Y	Y	DNK	N
G	Y	Y	SD	0	N	3	60	4	Y	N	N	N	DNK	N
H	Y	Y	Tauns	0	N	1	-	6	Y	N	N	Y	Y	Y
I	N	Y	SD	0	Y	4	-	2	Y	Y	n.a.	N	N	Y
J	N	Y	SD	0	N	6	-	4	N	N	Y	N	DNK	Y
K	Y	N	3	0	Y	3	48	12	Y	N	N	Y	DNK	N
L	Y	N	n.a.										-	Y
M	Y	N	n.a.										Y	N
N	N	N	n.a.										Y	Y
O	N	N	n.a.										DNK	Y

NRL: national TB reference laboratory, ICT: immunochromatographic test, Y: Yes, N: No, DNK: Did not know, n.a.: not applicable, Room: Room temperature, Tauns: Capilia TB assay (Tauns, Japan), SD: TB Ag MPT64 Rapid assay (SD Bio Standard Diagnostic, India/South Korea), BD: BD MGITTM TBc Identification Test (BD Diagnostics, USA).

1 List of countries eligible for negotiated pricing schemes for immunochromatic speciation tests as shown on the Foundation for Innovative New Diagnostics (FIND) website (http://www.finddiagnostics.org/about/what_we_do/successes/find-negotiated-prices/bactec-mgit.html).

2 Also mentioned Genotype MTBDRplus (Hain, Germany) and GeneXpert (Cepheid, USA).

3 Mentioned AccuProbe (GenProbe, USA).

### Adequacy of internal forecasting, stock management and storage procedures

In the ten TB reference laboratories using ICTs, it was mostly the laboratory manager or personnel that ordered ICTs from the manufacturer or local distributor (eight labs), as opposed to staff from the NTP (one lab) or the ministry of health (one lab). One NRL reported to have ICT stock-outs, occurring usually once every six months. This laboratory, where ministry of health staff ordered ICTs, complained about national procedures impeding effective test procurement. Normally, a new order was placed every 4.5 months and the number of tests requested was based on previous orders and sample load. In all except one NRL records were kept of test usage.

To get an idea of wastage, the questionnaire enquired about storage conditions and test handling. Seven NRLs stored their test kits between 2–8 degrees Celsius and three at room temperature, which was not monitored in two cases. Expired ICTs were disposed of as waste in two laboratories and used for testing in one, while four laboratories never experienced test expiry (three respondents did not know).

### Access to affordable diagnostic tests through negotiated pricing schemes

The NRL managers were asked about procurement pricing of ICTs for their laboratory network. Three out of nine (33%) were in possession of a commercial sales contract with a manufacturer. Assay prices as supplied by the same manufacturer could vary up to 100% between countries and ranged from $4 for a Capilia TB-Neo test to $18 for a BD MGITTM TBc Identification Test (only four countries indicated actual prices). Five countries indicated that they were eligible for FIND's negotiated pricing schemes for ICTs, but two of them could not be found on the online country list; two countries indicated to not be eligible, but were on the country list; and seven countries were not aware of their eligibility. Of the ten NRLs using ICTs, seven were based in countries eligible for FIND's negotiated pricing scheme set up with BD, but none of them used the BD MGITTM TBc Identification Test. When NRL managers were asked whether their laboratories paid the negotiated price for ICTs, they either did not know or provided no answer.

### Diagnostic algorithms in which ICTs are used

As indicated before, ten out of 15 (67%) NRLs were using ICTs for culture speciation. The majority of NRLs routinely performed other methods to identify bacteria of the MTB complex in cultures besides ICTs. All 15 NRLs performed smear microscopy on culture isolates to detect acid fast bacilli (AFB), of which 13 specifically looked for serpentine cords under the microscope (also called ‘cording’). Seven laboratories (47%) used biochemical methods and five (33%) used line probe assays to identify MTB complex for culture speciation, while six out of ten (60%) performed these tests in addition to ICTs. Table 3 shows a ranking of different speciation methods by the order in which they were routinely performed in the laboratory, with number 1 indicating the first test performed and number 5 the last test performed (two responses missing). It is clear that AFB microscopy of culture isolates – often in combination with looking for cording - was initially done by all NRLs, only to be followed by either ICTs, line probe assays and/or biochemical methods.

**Table 3:** Order in which different laboratory tests for identification of bacteria of the *Mycobacterium tuberculosis* complex in positive culture isolates are performed in national TB reference laboratories of 15 high endemic countries.

	Tests to identify TB culture isolates				
NRLs	Microscopy to detect AFB	Microscopy to detect cording	Immuno-chromato-graphic test	Line probe assay to detect MTB	Biochemical methods
A	1	2	3	-	-
B	1	1	2	3	4
C	1	2	3	-	-
D *	√	√	√	-	√
E	1	2	4	-	3
F	1	2	3	-	-
G	1	1	2	3	4
H	1	1	3	2	-
I *	√	√	√	-	√
J	1	-	2	-	-
K	1	2	-	3	-
L	1	2	-	3	-
M	1	1	-	-	2
N	1	2	-	-	-
O	1	-	-	-	2
**Average order**	**1.1 (14/13)**	**1.5 (17/11)**	**2.8 (22/8)**	**2.8 (14/5)**	**3.0 (15/5)**

NRL: national TB reference laboratory, AFB: acid fast bacilli, MTB: *Mycobacterium tuberculosis*.

* NRL indicated the type of tests performed, but not the order in which these tests were performed.

## Discussion

This survey intended to look into the access of NRL managers in 42 high endemic TB countries to gather information about retooling with ICTs for culture speciation, as well as their ability to effectively procure and utilize these tests. The fact that only 16 out of 42 (38%) NRL managers that were asked to participate in this survey responded to the questionnaire introduced a source of selection bias, which was the main limitation of this survey. Reasons for the low response rate could have been that email communications and questionnaires were in English only, thereby introducing a probable language barrier and source of misinterpretation bias. Considering the official WHO languages spoken by the contacted countries, 22 were English speaking and 20 were non-English speaking (ten Russian, four French, three Arabic, two Spanish, one Chinese). In total, eight out of 22 (36%) English speaking countries and eight out of 20 (40%) non-English speaking countries responded, including answers from five out of ten (50%) Russian speaking countries. This suggests that the low response rate could not only be attributed to a language issue. Given the efforts made to obtain valid contact details and follow up on responses by email, it is unclear what the exact reason is for the low response rate. This, combined with the fact that a quarter of NRL managers could not open electronic documents that were sent and half of them was not aware of WHO recommendations on liquid culture nor of their eligibility for financial support, strongly suggests the need to design and strengthen effective means of communication between NRLs and partners of the global laboratory network. As a consequence of the low response rate, no strong arguments can be made on the use of ICTs in high TB endemic countries based on quantitative results. With at least one questionnaire returned from every WHO region and from each type of high TB endemic country, this work therefore merely attempts to describe general findings and highlight possible causes for concern which warrant further investigation.

This survey showed that even though half of NRLs were unaware of the recommendation to use ICTs as described in WHO guidance documents, 80% of laboratories that performed liquid culture also used ICTs. And although half of them had no commercial sales contract for ICTs, they reported no major issues with test stock-outs, delivery time and remaining shelf-life that could affect the availability and accessibility of ICTs. When assessing affordability, half of NRLs were not aware of their eligibility for the FIND negotiated pricing agreement for ICT procurement and none of them was able to say whether their laboratory paid the negotiated price. Moreover, all NRLs using ICTs and eligible for favorable pricing for BD products used products from other suppliers and none reported test prices as low as the negotiated price. One respondent, who mistakenly thought his/her country was not eligible for negotiated pricing for ICTs, indicated that the high test costs were prohibitive for effective test procurement. Realizing that some of these NRLs received their ICTs directly from donor agencies or local NGO's and did not pay for the tests themselves, these findings may illustrate a missed opportunity for NRLs to request cheaper tests.

Another finding was that 60% of NRLs that used ICTs performed biochemical methods and/or line probe assays as additional tests to identify MTB complex in patient culture samples either before or after ICTs. Since ICTs are highly sensitive and specific for identifying MTB complex [4], [18], [19], performing biochemical methods and line probe assays seems like a duplication of efforts which needlessly increases workloads in laboratories. In addition, some countries specified that they used GenoType MTBDRPlus (Hain Lifescience GmbH, Germany), Accuprobe (GeneProbe, USA) and GeneXpert MTB/RIF (Cepheid, USA) tests for culture speciation, which use DNA isolation, amplification and detection to identify MTB complex in patient specimen [20], [21]. These tests are not recommended as initial culture speciation tests, because they are more technically demanding (Genotype MTBDRPlus and Accuprobe) and more expensive (GeneXpert MTB/RIF) than antigen-detecting ICTs. However, both GenoType MTBDRPlus and GeneXpert MTB/RIF tests also detect drug resistance and thereby provide an added benefit to ICTs. It is unclear whether WHO recommends that ICTs can be omitted in settings where all positive cultures are tested with drug resistance detecting assays.

Finally, none of the responding NRLs used ICTs as initial test for speciation of culture isolates. All NRLs first performed smear microscopy on culture isolates to detect AFB and most continued to investigate smears for cording, which is indicative for bacteria of the MTB complex like *M. tuberculosis*. A recent study of Chihota *et al.* found that an ICT using anti-MPB64 antibodies performed equally well in identifying culture isolates as microscopic cording, but the costs per identified positive culture was almost seven times higher [22]. NTMs are occasionally reported to show cording under the microscope, while some strains of the MTB complex do not show cording. However, especially in high TB endemic countries, the question should be asked why laboratories would increase their workload by performing both tests. While ICTs for speciation are preferred to standard biochemical tests and line probe assays for identification of MTB complex because they are more rapid and less costly, there is no apparent reason to prefer ICTs above microscopic cording except that no microscopy training is required. Future comparative studies on effectiveness and cost-effectiveness of ICTs and microscopic cording could provide more insight in the suitability of these tests for use in resource-constrained settings.

In conclusion, the low response rate of this survey together with NRLs managers' unawareness of global guidance, suggest a lack of effective communication between partners of the global laboratory network and NRLs. Half of the respondents was not aware of the WHO recommendation to use ICTs for culture speciation or the overall requirements for successful implementation, yet this did not negatively affect availability or accessibility of ICTs. If the possibility to negotiate lower prices for commercial products is communicated more successfully to NRLs by organizations involved in implementing TB diagnostics, TB tests will become more affordable to high endemic countries. The findings further raised a concern on the efficient use of ICTs to identify TB culture isolates in NRLs. Cheaper speciation methods like microscopic cording are often already part of routine laboratory practice and may perform just as well as ICTs. High TB endemic countries need to identify where the available technologies can be most usefully implemented and in what order. WHO can support these countries by providing additional guidance on how to best combine currently used and new diagnostic tools into long-term laboratory strategies.
